# Enhancement of *SMN2* Exon 7 Inclusion by Antisense Oligonucleotides Targeting the Exon

**DOI:** 10.1371/journal.pbio.0050073

**Published:** 2007-03-13

**Authors:** Yimin Hua, Timothy A Vickers, Brenda F Baker, C. Frank Bennett, Adrian R Krainer

**Affiliations:** 1 Cold Spring Harbor Laboratory, Cold Spring Harbor, New York, United States of America; 2 Isis Pharmaceuticals, Carlsbad, California, United States of America; National Cancer Institute, United States of America

## Abstract

Several strategies have been pursued to increase the extent of exon 7 inclusion during splicing of *SMN2 (survival of motor neuron 2)* transcripts, for eventual therapeutic use in spinal muscular atrophy (SMA), a genetic neuromuscular disease. Antisense oligonucleotides (ASOs) that target an exon or its flanking splice sites usually promote exon skipping. Here we systematically tested a large number of ASOs with a 2′-*O*-methoxy-ethyl ribose (MOE) backbone that hybridize to different positions of *SMN2* exon 7, and identified several that promote greater exon inclusion, others that promote exon skipping, and still others with complex effects on the accumulation of the two alternatively spliced products. This approach provides positional information about presumptive exonic elements or secondary structures with positive or negative effects on exon inclusion. The ASOs are effective not only in cell-free splicing assays, but also when transfected into cultured cells, where they affect splicing of endogenous *SMN* transcripts. The ASOs that promote exon 7 inclusion increase full-length SMN protein levels, demonstrating that they do not interfere with mRNA export or translation, despite hybridizing to an exon. Some of the ASOs we identified are sufficiently active to proceed with experiments in SMA mouse models.

## Author Summary

Spinal muscular atrophy (SMA) is a severe genetic disease that causes motor-neuron degeneration. SMA patients lack a functional *SMN1 (survival of motor neuron 1)* gene, but they possess an intact *SMN2* gene, which though nearly identical to *SMN1,* is only partially functional. The defect in *SMN2* gene expression is at the level of pre-mRNA splicing (skipping of exon 7), and the presence of this gene in all SMA patients makes it an attractive target for potential therapy. Here we have surveyed a large number of antisense oligonucleotides (ASOs) that are complementary to different regions of exon 7 in the *SMN2* mRNA. A few of these ASOs are able to correct the pre-mRNA splicing defect, presumably because they bind to regions of exon 7 that form RNA structures, or provide protein-binding sites, that normally weaken the recognition of this exon by the splicing machinery in the cell nucleus. We describe optimal ASOs that promote correct expression of *SMN2* mRNA and, therefore, normal SMN protein, in cultured cells from SMA patients. These ASOs can now be tested in mouse models of SMA, and may be useful for SMA therapy.

## Abstract 3

Mutations in*SMN1* cause spinal muscular atrophy; a nearly identical gene is not functional, but becomes functional in vitro and in vivo after addition of antisense oligos.

## Introduction

Spinal muscular atrophy (SMA), the most common genetic cause of infant mortality, is an autosomal recessive neuromuscular disease characterized by progressive loss of α-motor neurons in the anterior horns of the spinal cord, leading to limb and trunk paralysis and atrophy of voluntary muscles. Based on the severity and age of onset, SMA is clinically subdivided into types I, II, and III (MIMs 253300, 253550, and 253400), with type I being the most severe [1]. SMA has an incidence of approximately one in 6,000 live births, and a carrier frequency of one in 40.

Loss of function of the *survival of motor neuron 1 (SMN1)* gene is responsible for SMA [2]. Mice with homozygous *SMN1* disruption display massive cell death during early embryogenesis [3]. SMN protein is ubiquitously expressed and is mainly localized in the cytoplasm and in nuclear “gems” [4]. Multiple SMN-interacting partners have been identified, suggesting the involvement of SMN in various cellular processes, such as transcription, mRNA transport, and assembly of ribonucleoprotein particles (RNPs), including small nuclear RNPs (snRNPs), small nucleolar RNPs (snoRNPs), and stress granules [5–8].

Humans have an extra *SMN* gene copy, designated *SMN2*. Both *SMN* genes reside within a segmental duplication on Chromosome 5q13 as inverted repeats [2]. *SMN1* and *SMN2* are almost identical, except for 11 nucleotide substitutions: seven in intron 6, two in intron 7, one in coding exon 7 (a translationally silent C to T transition, relative to *SMN1*), and one in non-coding exon 8 [9]. *SMN2* is a prototypical example of alternative splicing caused by a single nucleotide substitution in the affected exon. Exon 7 is efficiently included in spliced mRNA from the *SMN1* gene; however, the silent C6T transition in *SMN2* exon 7, which weakens the recognition of the upstream 3′ splice site [10], results in significant skipping of this exon during pre-mRNA splicing. The C6T transition abrogates a splicing factor 2/alternative splicing factor (SF2/ASF)-dependent exonic splicing enhancer (ESE); this SF2/ASF heptamer motif is present in *SMN1* exon 7 (nucleotides +6 to +13, CAGACAA) and supersedes the inhibitory effect of heterogeneous nuclear ribonucleoprotein (hnRNP) A1 [11–13]. As a result, *SMN2* encodes mostly the exon 7–skipped protein isoform (SMNΔ7), which is unstable, mislocalized, and at best, only partially functional [14–16]. Although the small amount of full-length SMN protein derived from *SMN2* is not sufficient to fully compensate for loss of *SMN1,* it is essential for viability in the absence of *SMN1,* and is an important disease modifier: in both SMA patients and mouse models, there is an inverse relationship between *SMN2* copy number and disease severity [17,18]. These properties define *SMN2* as an ideal therapeutic target for potential treatment of SMA.

Antisense technology was initially employed to down-regulate gene expression by targeting mRNA to induce its degradation or block its translation [19]. Advances in antisense chemistry allowed this technology to be applied as a powerful tool to manipulate pre-mRNA splicing. Modification of the base, sugar, or phosphodiester structure of oligonucleotides generates highly stable molecules with not only high affinity for RNA targets, but also resistance to various nucleases, including RNase H (which cleaves RNA in RNA/DNA hybrids). The Kole laboratory pioneered the use of antisense oligonucleotides (ASOs) to correct aberrant splicing by targeting pre-mRNA, demonstrating both in vitro and in vivo that ASOs can restore correct expression of a defective β-globin gene by blocking the cryptic splice sites generated by intronic mutations that cause β-thalassemia [20,21]. Recently, ASO applications to redirect pre-mRNA splicing were extended to genes with therapeutic relevance to cancer or other diseases [22–24]. ASOs have been employed to promote skipping of non-essential exons, so as to restore the translational reading frame and suppress the effects of various deletion, nonsense, and frameshifting alleles of the dystrophin gene *(DMD)* [25–27]. Although it is now relatively straightforward to block the use of specific exons by targeting the corresponding 5′ or 3′ splice sites or empirically selected sequences within the exon, it remains much more challenging to find suitable antisense targets for promoting exon inclusion.

Several antisense approaches have been documented to promote *SMN2* exon 7 splicing. One approach is based on the notion that there is a competition between the 3′ splice sites of exons 7 and 8 for pairing with the 5′ splice site of exon 6 [10], so impairing the recognition of the 3′ splice site of exon 8 should favor exon 7 inclusion. Increased *SMN2* exon 7 inclusion and full-length SMN protein were indeed observed in HeLa cells transfected with a modified U7 small nuclear RNA (snRNA) complementary to the intron 7/exon 8 junction [28]. Another approach, called TOES (targeted oligonucleotide enhancer of splicing) [29], relies on bifunctional ASOs composed of one segment complementary to exon 7 and a non-complementary tail consisting of RNA sequences with ESE motifs recognized by a serine/arginine-rich (SR) protein. One such bifunctional ASO stimulated *SMN2* exon 7 inclusion and led to significant restoration of gem number—an indicator of SMN protein increase—in SMA-patient fibroblasts [30]. Moreover, an in vivo delivery system was recently developed for expression of bifunctional ASOs in an adeno-associated virus vector [31].

Strong intronic splicing silencers (ISSs) represent ideal antisense targets; however, if present, they are usually inconspicuous within large introns. An ISS in intron 6 was identified in the context of an *SMN2* minigene, and targeting this inhibitory element with an ASO increased exon 7 inclusion in transiently transfected COS-7 cells [32]. An intron 7 ISS, located immediately downstream of the 5′ splice site of exon 7, was recently reported; an ASO against this silencer efficiently restored exon 7 inclusion of *SMN2* and increased SMN protein levels in SMA-patient–derived cells [33].

The ESSENCE (exon-specific splicing enhancement by small chimeric effectors) method, which was developed to rescue disease-associated exon skipping or modulate alternative splicing, employs small chimeric effectors designed to emulate SR proteins [34,35]. A synthetic ESSENCE molecule comprises two portions: an antisense moiety complementary to a target exon; and a minimal RS domain peptide similar to the splicing activation domain of SR proteins. We described several ESSENCE compounds that significantly promote *SMN2* exon 7 inclusion in a cell-free splicing assay [34]. Interestingly, the antisense moiety alone also significantly stimulates exon 7 inclusion, although with approximately 8-fold less potency compared to ESSENCE compounds, suggesting the existence of an exonic splicing silencer (ESS) in exon 7 [34]. This observation highlighted the possibility of further optimization of the antisense moiety of ESSENCE compounds to enhance their potency. Here, we used two-step ASO walks along *SMN2* exon 7 to define position-dependent effects of ASO-binding sites. With the first coarse whole–exon 7 walk, we identified two ASO target regions separated by a central core sequence, and with the second high-resolution walks, we identified optimal ASOs against each target region. The two best ASOs strongly promoted exon 7 inclusion during *SMN2* pre-mRNA splicing in three different splicing assays, and increased SMN protein levels in cultured cells, including SMA type I fibroblasts. Thus, appropriate ASOs targeting a coding exon can increase protein levels by promoting exon inclusion and not interfering with translation. Most importantly, the effective ASOs should be suitable for studies in animals and to further optimize bifunctional or peptide-linked ASOs for better efficacy in rescuing *SMN2* splicing and full-length protein expression.

## Results

### ASO Walk along *SMN2* Exon 7

Previously we reported that one PNA (peptide nucleic acid) ASO complementary to positions +7 to +18 of *SMN2* exon 7 stimulates exon 7 inclusion during splicing in vitro [34]. This unexpected observation suggested that the ASO blocked one or more putative ESSs—either a specific motif or a secondary structure that impairs the recognition of exon 7. To pinpoint the exact locations of these presumptive elements, we used a systematic ASO walk along the entire length of *SMN2* exon 7. Oligonucleotides with a 2′-*O*-methoxy-ethyl ribose (MOE) phosphodiester backbone were used. This backbone modification imparts a very high affinity for targeted mRNA, resistance to both exo- and endonucleases, and does not support cleavage of hybridized mRNA by RNase H [36,37]. We synthesized nine MOE 15-mer ASOs complementary to the 54-nucleotide (nt) exon 7; beginning with the first position of the exon, the overlapping ASOs provide coverage in 5-nt increments from 5′ to 3′, with a last step of 4 nt to the last position of the exon (Figure 1A, Table 1). An unrelated oligonucleotide, 00–00, was used as a negative control (Table 1).

**Figure 1:**
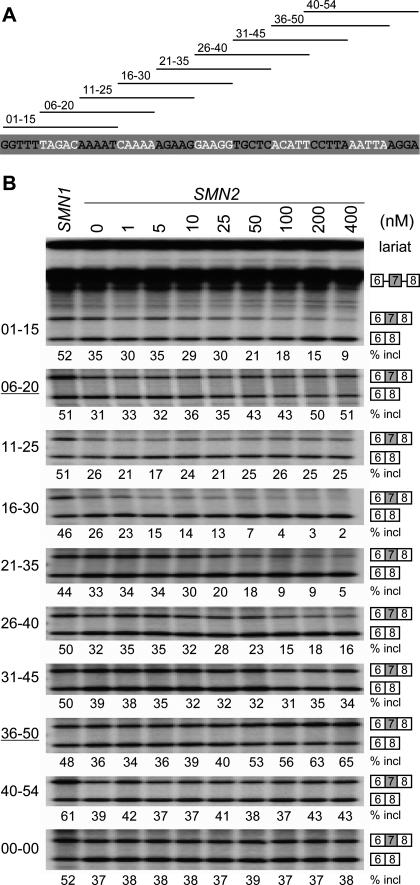
Initial ASO Walk along Exon 7, Assayed by In Vitro Splicing (A) Schematic representation of the binding sites for the nine MOE ASOs used in the initial exon 7 walk. The position of complementarity of each ASO along exon 7 is indicated by a horizontal line. (B) Effect of ASOs on in vitro splicing of *SMN2* minigene-derived pre-mRNA. Each ASO was tested at the indicated concentrations, and *SMN1* pre-mRNA was used as a positive control. The radiolabeled RNAs were analyzed by denaturing PAGE and autoradiography. The diagrams on the right indicate the mobilities of the various RNA species. The percentage of exon 7 inclusion in each lane was calculated as described in Materials and Methods, and is indicated below each autoradiogram. The two ASOs that promote exon 7 inclusion are underlined.

**Table 1:**
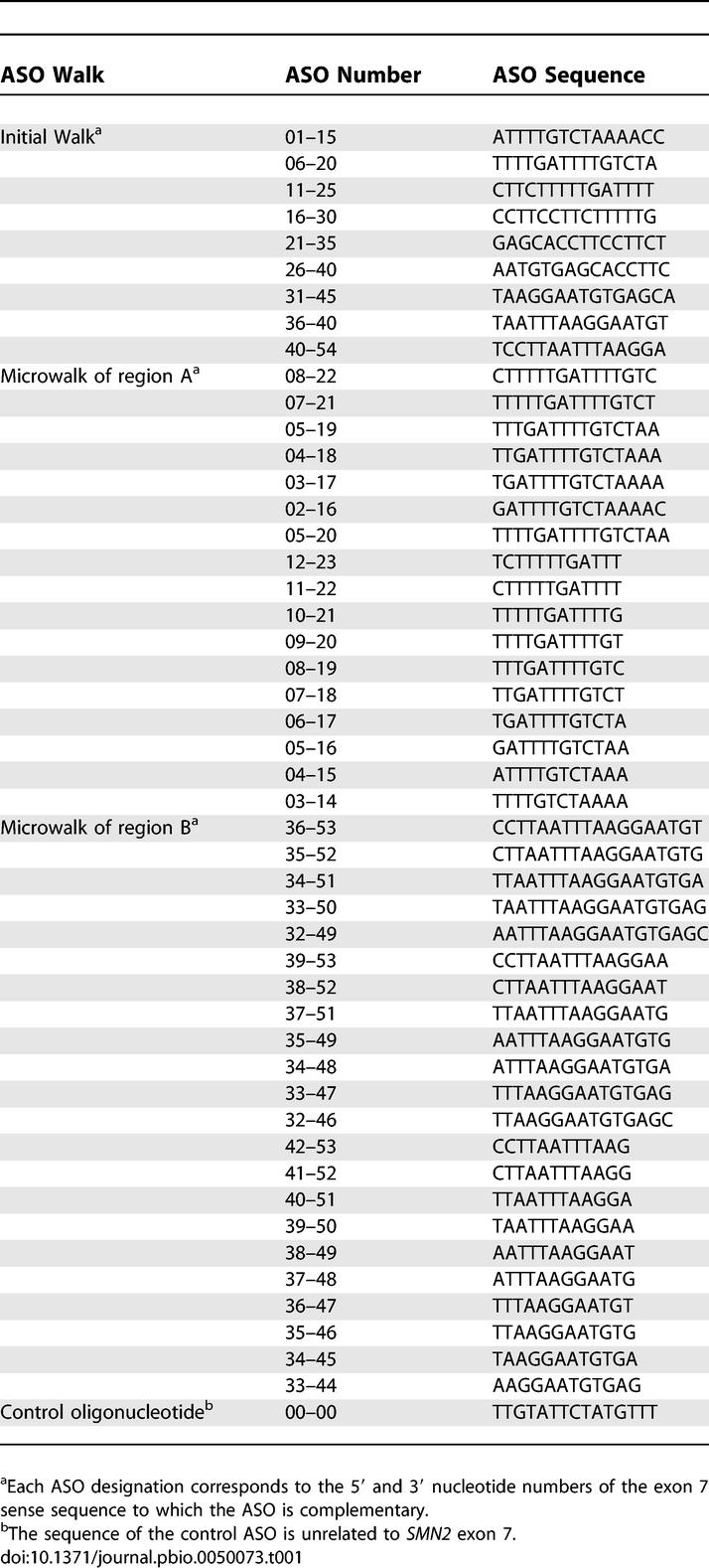
MOE ASOs Used in the Two-Step Walks, and Their Exon 7 Targets

We first evaluated these ASOs by in vitro splicing in HeLa cell nuclear extract with a radiolabeled *SMN2* minigene transcript [11]. Although the precise extent of exon 7 inclusion varies between extracts or depending on the precise reaction conditions, the difference between *SMN1* and *SMN2* is highly reproducible. We tested eight different concentrations of each ASO, from 1 to 400 nM, compared to no ASO. As shown in Figure 1B, the nine ASOs had different effects on splicing of the *SMN2* pre-mRNA. Four ASOs, 01–15, 16–30, 21–35, and 26–40, strongly inhibited exon 7 inclusion; ASOs 11–25 and 31–45 slightly inhibited exon 7 inclusion; and ASO 40–54 and the control oligonucleotide 00–00 had no effect on alternative splicing of *SMN2* exon 7. Interestingly, we found two ASOs, 06–20 and 36–50, that significantly stimulated exon 7 inclusion in the cell-free splicing assay, starting at a concentration of approximately 50 nM.

ASO 01–15 targets the exonic portion of the 3′ splice site, whereas ASOs 11–25, 16–30, 21–35, and 26–40 target a central region that comprises a transformer 2 beta 1 protein (Tra2β1)-binding motif in *SMN* exon 7 [38]. Both elements are required for exon 7 inclusion, which can account for the negative effects of these ASOs. ASO 31–45 has a 10-nt overlap with the stimulatory ASO 36–50, yet it had a negative effect on splicing of exon 7, suggesting that there is a motif upstream of the ASO 36–50-binding site that is also important for exon 7 inclusion. ASO 40–54 targets the exonic portion of the exon 7 5′ splice site; however, it is essentially neutral. We offer two explanations for this observation: first, the exonic portion of this 5′ splice site deviates from the consensus sequence, and thus ASO 40–54 might not affect U1 snRNA base-pairing; second, ASO 40–54 has a strong predicted stem-loop structure (unpublished data) and might not bind efficiently to its complementary sequence. It is not surprising that ASO 06–20 had a positive effect, because it encompasses the sequence of the antisense PNA we reported previously [34]; however, ASO 36–50, which is complementary to nucleotides +36 to +50 of exon 7, defines an additional region in *SMN2* exon 7 that can be targeted to increase exon 7 inclusion.

To determine whether the ASO-mediated stimulation of exon 7 inclusion can take place in cells, we co-transfected plasmid pCI-SMN2 with each of the nine MOE ASOs into HEK293 cells by electroporation. At the same time, we co-transfected plasmid pBabe Puro and selected the transfected cells by treatment with puromycin. Two days after transfection, we analyzed the transiently expressed RNA by RT-PCR (Figure 2A). Each of the ASOs gave similar effects on splicing of exon 7 in the minigene pre-mRNA as observed by in vitro splicing (Figure 1B), except for two ASOs, 11–25 and 31–45. These two ASOs, especially the latter one, resulted in robust inhibition of exon 7 inclusion in vivo, whereas only slight effects were observed in vitro.

**Figure 2:**
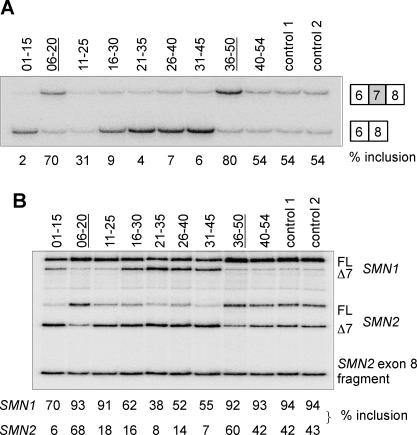
In Vivo Validation of the Initial ASO Walk Each ASO at a concentration of 10 μM and 2.5-μg pBabe Puro with or without 5-μg pCI-SMN2 were transfected by electroporation, and transfected cells selected with 2-μg/ml puromycin for 20 h. Two days after transfection, cells were collected for total RNA preparation, and RT-PCR was performed to analyze *SMN2* pre-mRNA splicing patterns. The PCR products were labeled by incorporation of α-^32^P-dCTP. The ASOs that promote exon inclusion are underlined. Control 1: unrelated oligonucleotide 00–00; control 2: buffer. (A) The nine ASOs were co-transfected with pCI-SMN2, and the PCR products analyzed by 8% native PAGE. (B) The effects of the nine ASOs were analyzed with transcripts from the endogenous *SMN2* gene in HEK293 cells. RT-PCR products were digested with DdeI to distinguish *SMN1* from *SMN2* by 6% native PAGE. The percentage of exon 7 inclusion in each lane is indicated below each autoradiogram. FL, full-length mRNA; Δ7, exon 7–deleted mRNA.

We next tested the effect of the exon 7 ASOs on splicing of transcripts from the endogenous *SMN* genes in HEK293 cells. This time, only the ASOs and plasmid pBabe Puro were electroporated into the cells. The transfected cells were puromycin-selected as above, and RNA samples were collected 2-d post-transfection. After RT-PCR, we digested the cDNA with DdeI to distinguish *SMN2* mRNA from *SMN1* mRNA [39,40]. As expected, the effects of these MOE ASOs on splicing of transcripts from the endogenous *SMN2* gene were very similar to those we observed with the *SMN2* minigene (Figure 2B). The only notable difference between the two assays was in the relative efficacy of the two positive ASOs, 06–20 and 36–50. ASO 06–20 was less efficient in stimulating exon 7 inclusion than ASO 36–50 in the in vitro splicing and minigene splicing assays, but it was reproducibly more efficient than ASO 36–50 in the endogenous *SMN2* splicing assay.

On the basis of the above results obtained with the three splicing assays, we conclude that exon 7 comprises three distinct regions: a core sequence that is essential for exon 7 inclusion, and two flanking regions that correspond to putative ESSs. These two inhibitory regions correspond to the binding sites for ASOs 06–20 and 36–50, and are designated region A and region B, respectively.

### High-Resolution Microwalks within Regions A and B

Having roughly delineated regions A and B as effective antisense targets to stimulate exon 7 inclusion, we sought to define their boundaries more precisely by identifying ASOs with optimal sequences and lengths, and thus maximize exon 7 inclusion. To this end, we designed 39 new ASOs: 17 ASOs of length 12, 15, or 16 nt, spanning 21 nt within or overlapping region A (microwalk A), and 22 ASOs of length 12, 15, or 18 nt, spanning 22 nt within or overlapping region B (microwalk B) (Figure 3A). The sequences of all 39 ASOs are shown in Table 1. We first screened all these MOE ASOs using the in vitro splicing assay and a concentration of 100 nM for each ASO.

**Figure 3:**
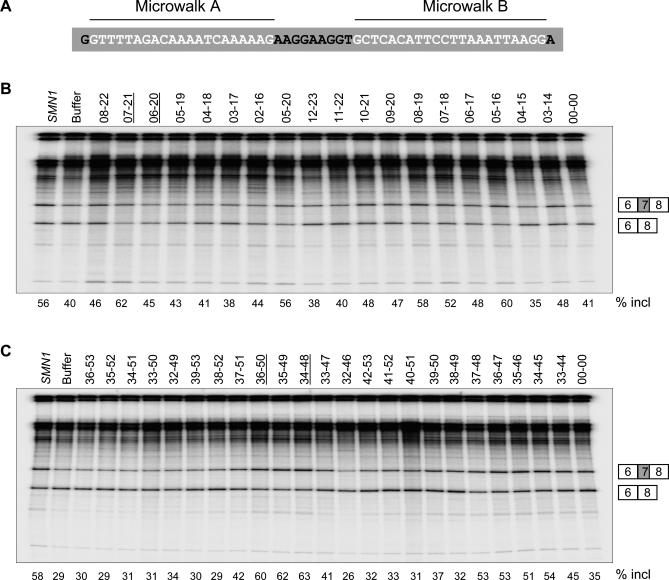
Two ASO Microwalks Assayed by In Vitro Splicing (A) Nucleotides in white show the two microwalk regions. (B) Seventeen new ASOs were screened in microwalk A. The original ASO (06–20) and the improved ASO (07–21) are underlined. (C) Twenty-two new ASOs were screened in microwalk B. The original ASO (36–50) and the improved ASO (34–48) are underlined. *SMN2* minigene pre-mRNA was spliced in vitro as in Figure 1, in the presence of each ASO at a concentration of 100 nM. The percentage of exon 7 inclusion in each lane is indicated below each autoradiogram.

The 15-mer ASO 07–21 was identified as the most effective one in the case of microwalk A (Figure 3B). Its target site is shifted downstream by one nucleotide, relative to the target site of the original ASO 06–20. In the presence of this new ASO, the extent of exon 7 inclusion increased to 62%, representing a significant improvement over the original 15-mer ASO 06–20, which gave 45% exon 7 inclusion in this experiment. Microwalk B generated another improved ASO, the 15-mer ASO 34–48, though at this concentration, it gave only a slight improvement compared to the original ASO (Figure 3C and unpublished data). The target site for this new ASO is shifted upstream by two nucleotides, relative to the target site of the original 15-mer ASO 36–50. We further characterized the two improved high-resolution walk ASOs by titration in the in vitro splicing assay. Concentrations ranging from 1 to 400 nM of ASO 07–21 or 34–48 were tested and compared with the original ASO 06–20 or 36–50 identified in the coarse walk. The splicing data and dose-response curves are shown in Figure 4. The median effective concentration (EC50) values for the two original ASOs 06–20 and 36–50 were 56 nM and 47 nM, respectively, whereas the EC50 values for the improved ASOs 07–21 and 34–48 were reduced to 16 nM and 13 nM, respectively.

**Figure 4:**
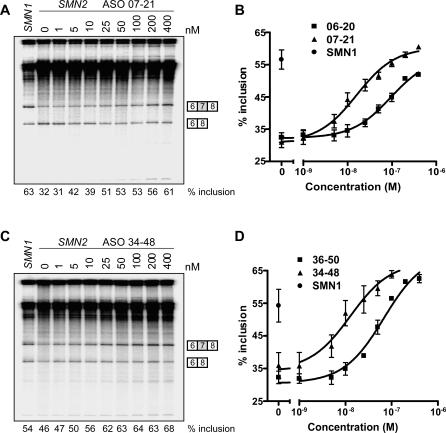
Dose-Response Analysis with the Two Improved ASOs Using In Vitro Splicing ASOs 07–21 (A) and 34–48 (C) were tested at the indicated concentrations. In vitro splicing was carried out as in Figure 1. The percentage of exon 7 inclusion in each lane is indicated below each autoradiogram. (B) and (D) Sigmoidal curves were plotted to compare the dose-response effects of the high resolution–walk ASOs (A) and (C) with the initial-walk ASOs (from Figure 1) using the data from three independent experiments. The error bars show standard deviations.

The effects of the two groups of microwalk ASOs were further examined in vivo, first with the minigene pCI-SMN2 and then with the endogenous *SMN1/2* genes, as described above. Each ASO, at a concentration of 10 μM, with or without the *SMN2* minigene plasmid, was electroporated into HEK293 cells (Figure 5A). ASO 07–21 stimulated exon 7 inclusion during splicing of the *SMN2* minigene pre-mRNA more effectively than ASO 06–20. As expected, ASO 07–21 also potently stimulated exon 7 inclusion during splicing of the endogenous *SMN2* pre-mRNA (Figure 5B). Similarly, ASO 34–48 robustly increased the inclusion of exon 7 for both the *SMN2* minigene and the endogenous *SMN2* gene transcripts (Figure 5C and 5D). The effects of all the antisense molecules on exon 7 splicing were generally consistent between in vivo minigene and endogenous gene assays. Compared to the in vitro splicing assay, the two in vivo splicing assays appeared more sensitive in terms of the stimulatory or inhibitory effects on exon 7 inclusion caused by the tested ASOs. In addition, some ASOs gave inconsistent effects between the in vitro assay and the two in vivo assays: three ASOs in microwalk A (08–22, 02–16, and 09–20) and three ASOs in microwalk B (33–47, 34–45, and 33–44) all slightly promoted exon 7 inclusion in vitro, but more or less inhibited exon 7 inclusion in vivo; ASOs 12–23, 35–52, 34–51, 33–50, and 32–49 showed neutral or negligible effects on exon 7 inclusion in vitro, but inhibited exon 7 inclusion in vivo to some extent; and finally, ASO 38–49 showed no effect on exon 7 inclusion in vitro or with the endogenous gene, but promoted exon 7 inclusion in the minigene co-transfections.

**Figure 5:**
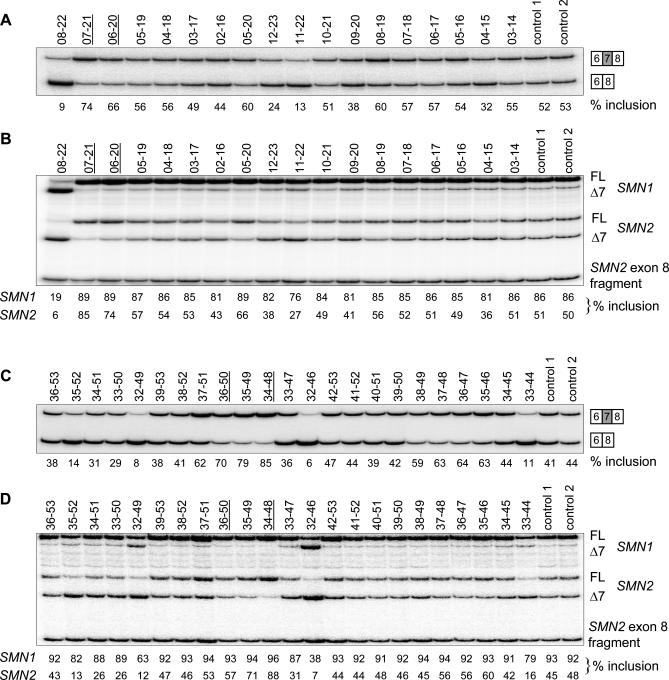
In Vivo Validation of the Two ASO Microwalks The effects of the 17 microwalk A ASOs were examined with the *SMN2* minigene expressed in HEK293 cells (A) or with the endogenous *SMN2* gene in HEK293 cells (B). The effects of the 22 microwalk B ASOs were examined with the *SMN2* minigene expressed in HEK293 cells (C) or with the endogenous *SMN2* gene in HEK293 cells (D). The in vivo splicing assays were carried out as in Figure 2. Control 1: unrelated oligonucleotide 00–00; control 2: buffer. The percentage of exon 7 inclusion in each lane is indicated below each autoradiogram.

The microwalk data obtained from the two in vivo assays not only verified the presence of a central core sequence, but also established the precise boundaries between the core sequence and regions A and B on either side of it. In microwalk A, the target site for the improved ASO 07–21 extends to three consecutive As of the proposed Tra2β1-recognition sequence (AAAGAAGGA) [38], suggesting that this portion of the Tra2β1-dependent ESE is not critical for binding of the protein. This interpretation is consistent with the finding that mutating the triple As to triple Us does not abrogate Tra2β1 recognition [38]. The triple As mark the 3′ boundary of region A; extending the walk farther downstream causes strong exon 7 skipping, as in the cases of ASOs 08–22, 12–23, and 11–22 (Figure 5A and 5B), presumably because of interference with Tra2β1 binding. In microwalk B, ASOs that bind farther upstream of the improved ASO 34–48, such as 33–47, 32–46, and 33–44, caused a strong reduction in exon 7 inclusion (Figure 5C and 5D). These three ASOs are complementary to exon 7 with a 5′-most boundary at +33C and +32G, respectively, suggesting that this GC dinucleotide is part of an element or structure important for exon 7 recognition. This observation is also consistent with the in vivo effect of ASO 31–45 in the initial exon 7 walk (Figure 2). The combined data from the coarse- and high-resolution antisense walks point to the existence of a core sequence in the middle of exon 7, from +22 to +33, that is essential for exon 7 inclusion.

The microwalk data obtained from the two in vivo assays are also useful for mapping both the 5′ boundary of region A and the 3′ boundary of region B. In microwalk A, ASO 04–18 displayed the least stimulatory effects among the 15-mer ASOs; other ASOs that target further upstream sequences were either neutral or inhibitory. These results define the boundaries of region A as +4 to +21. In microwalk B, the 15-mer ASOs 39–53 and 38–52, whose 3′-most complementary-sequence boundaries are +53G and +52G, respectively, were essentially neutral; all other 15-mer ASOs, except 33–47 and 32–46, which partly target the central core sequence, had stimulatory effects. Therefore, nucleotides +34 to +51 define the boundaries of region B. The results obtained with 12-mer ASOs are generally consistent with those obtained with 15-mer ASOs, except for ASO 04–15, which targets region A, but was unexpectedly inhibitory with respect to exon 7 inclusion, and ASO 09–20, which also targets region A, but was inhibitory in the in vivo assays.

### Dose-Response and Kinetic Analysis of ASO 07–21 and ASO 34–48

The two high-resolution walk exon 7 ASOs were further characterized with respect to their effects on splicing of the endogenous *SMN1/2* genes in HEK293 cells. We first conducted dose-response experiments with each ASO, using a concentration range from 0.2 to 20 μM for electroporation. Although we do not know the intracellular concentration of each ASO after electroporation, inclusion of exon 7 in both *SMN1* and *SMN2* transcripts showed a clear dependence on the dose of each ASO (Figure 6A and 6B). For both ASOs, a significant increase in exon 7 inclusion was already noticeable at a starting concentration of 0.2 μM; at 20 μM ASO, *SMN2* exon 7 inclusion reached 87%–89%, compared to 86%–87% *SMN1* and 50%–51% *SMN2* exon 7 inclusion in the untreated cells. After treatment with ASO 07–21 or ASO 34–48, the exon 7 inclusion level of the *SMN1* gene transcripts rose to 93%–95%, indicating that these two ASOs affect the recognition of sequence elements or secondary structures present in transcripts from both *SMN* genes.

**Figure 6:**
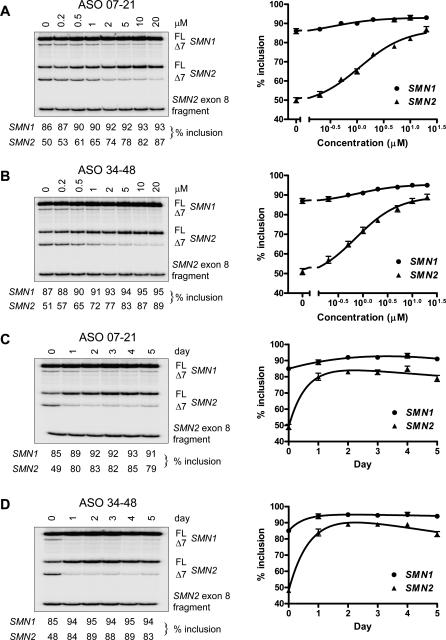
Dose-Response and Time-Dependence Analysis with the Two Improved ASOs for Splicing of Endogenous *SMN1/2* Pre-mRNAs ASOs 07–21 (A) and 34–48 (B) were transfected into HEK293 cells, using starting concentrations from 0 to 20 μM for electroporation. Dose-response curves for ASOs 07–21 and 34–48 were plotted on the right using the data obtained from three independent experiments. Error bars indicate standard deviations. ASOs 07–21 (C) and 34–48 (D) at a concentration of 10 μM were transfected into cells by electroporation. Cells were harvested daily for 5 d to prepare total RNA for splicing analysis as in Figure 2. Exponential-decay curves were plotted on the right using the data obtained from three independent experiments. Error bars indicate standard deviations. The percentage of *SMN1* and *SMN2* exon 7 inclusion in each lane is indicated below each autoradiogram.

Next, we analyzed the effects of the two ASOs over time, as a measure of their intracellular stability. We transfected HEK293 cells by electroporation with each ASO at 10 μM concentration, and total RNA samples from parallel transfections were collected daily at 1–5 d post-transfection. Robust effects were already apparent on the first day and reached a plateau by the second day (Figure 6C and 6D). The strong effects persisted for at least 5 d, though a slight decline of exon 7 inclusion level was observed, which might partly reflect dilution of the ASOs as the cells divided.

### Effect of ASO 07–21 and ASO 34–48 at the Protein Level

Both ASO 07–21 and ASO 34–48 strikingly increased the amount of full-length mRNA expressed from the endogenous *SMN1/2* genes. However, because the ASOs might remain associated with exon 7 in the spliced mRNA, they could potentially interfere with mRNA export and/or translation. Therefore, we sought to determine whether the stimulatory effect of these ASOs on *SMN1* exon 7 inclusion results in increased full-length SMN protein. We could not accurately determine this in HEK293 cells, because of the high levels of identical SMN protein expressed from the *SMN1* gene. Therefore, we generated another *SMN2* minigene construct, designated pEGFP-SMN2. This minigene consists of an N-terminal enhanced green fluorescent protein (EGFP), a short cloning-site sequence, a hemagglutinin epitope tag (HA tag), *SMN* exon 6, a shortened intron 6 (same as in minigene pCI-SMN2), exon 7, intron 7, and the 5′ end of exon 8 (75 nt) at the C-terminus (Figure 7A). When the pre-mRNA from this minigene is spliced, it generates two mRNAs via exon inclusion or skipping. Translation of the exon 7–included mRNA gives a larger protein of 308 amino acids (designated P-Incl), comprising EGFP, seven amino acids (SGLRSRE) derived from the cloning site, the HA tag, and exons 6 and 7 of SMN. The exon 7–skipped mRNA is translated into a smaller protein of 296 amino acids (designated P-Excl), comprising EGFP, SGLRSRE, the HA tag, exon 6, and four amino acids (EMLA) from exon 8 (Figure 7A). Both proteins can be detected with anti-HA antibody. Before proceeding to the protein-level analysis, we first verified the increase in exon 7 inclusion for the minigene pEGFP-SMN2 after electroporation with ASO 07–21 or 34–48. Compared with the endogenous *SMN2* gene, minigene pEGFP-SMN2 favors the exon 7–skipped mRNA isoform. The extent of exon 7 inclusion was about 28% without any treatment; however, after treatment with 10 μM ASO 07–21 or 34–48, exon 7 inclusion rose to 74% or 85%, respectively (Figure 7B). Protein samples were generated 3 d after the cells were co-transfected via electroporation with the minigene construct and either ASO at 10 or 30 μM concentration. Surprisingly, using mouse monoclonal anti-HA antibody, we only detected the exon-included protein product, P-Incl, with an apparent mass of approximately 38 kDa (Figure 7C). The effects of both ASOs were dose dependent, and after the treatment with 30 μM ASO, P-Incl reached almost the same level as observed with the *SMN1* minigene.

**Figure 7:**
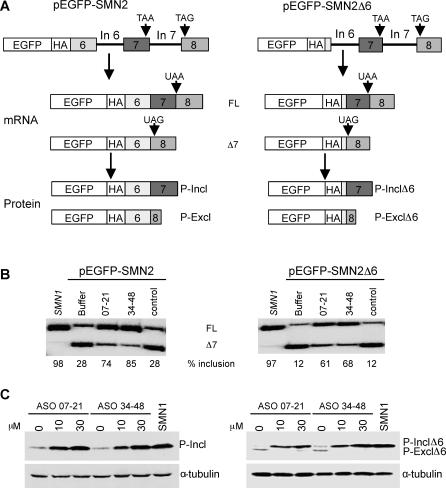
Effect of ASOs 07–21 and 34–48 at the mRNA and Protein Levels, Measured with Minigene Reporters Either ASO, at a concentration of 10 or 30 μM, together with a minigene reporter plasmid, was electroporated into HEK293 cells as in Figure 2. Three days after transfection and puromycin selection, cells were harvested to generate total RNA and protein samples. (A) Diagram of the reporter constructs. pEGFP-SMN2Δ6 lacks most of exon 6, compared to pEGFP-SMN2. The alternatively spliced mRNAs are designated E7-Incl and E7-Excl, and the corresponding protein products are P-Incl and P-Excl. The natural stop codons in exons 7 and 8 are shown. (B) Total RNA samples from cells treated with 10 μM ASO were analyzed by radioactive RT-PCR with EGFP- and exon 8–specific primers. Controls include the unrelated ASO 00–00, buffer only, and an *SMN1* version of the reporter minigene. The percentage of the exon-7–included isoform is indicated below each lane. (C) The proteins expressed from the reporters were detected by Western blotting with a monoclonal antibody against the HA tag; α-tubulin was detected with a monoclonal antibody as a loading control. Although exon-7–excluded mRNA expressed from the pEGFP-SMN2 plasmid was detected (B), the corresponding protein was apparently unstable (C).

We were not able to detect any P-Excl protein produced from the *SMN2* Δ7 mRNA transcript; we believe this is due to protein instability. SMNΔ7, the unstable protein isoform derived from the *SMN1/2* genes, and P-Excl share the same C-terminus, corresponding to the peptide coded by exon 6 plus four amino acids derived from exon 8; it is possible that these sequences, when present close to the C-terminus, induce degradation of the protein. To overcome this problem, we constructed another *SMN2* minigene, pEGFP-SMN2Δ6; this construct is similar to pEGFP-SMN2, but lacks most of exon 6, except for the last 9 nt, so as to maintain an intact natural 5′ splice site (Figure 7A). This minigene encodes two protein isoforms: the exon 7–included one (P-InclΔ6; 274 amino acids) and the exon 7–excluded one (P-ExclΔ6; 262 amino acids). Using this minigene, we again tested ASO 07–21 or 34–48 in HEK293 cells; significant increases in both exon 7–included mRNA and protein were observed (Figure 7B and 7C). This time, using anti-HA antibody, we detected two polypeptides from pEGFP-SMN2Δ6, with sizes of about 32 kDa and 30 kDa, and with the latter giving a stronger signal. After treatment with the ASOs, the P-ExclΔ6 band disappeared, whereas the P-InclΔ6 band became considerably stronger and comparable in intensity to the *SMN1* minigene product. To control for sample loading differences, the same blots were reprobed with anti–α-tubulin monoclonal antibody. These results clearly demonstrate that ASOs targeting a coding exon are able to promote exon inclusion at the mRNA level, and the resulting mRNA can be translated into protein.

### Effects of ASO 07–21 and ASO 34–48 in SMA-Patient Fibroblast Cells

To test whether the in vivo results in human HEK293 cells can be extended to SMA-patient cells, SMA type I 3813 fibroblasts were transfected with either ASO at 100 nM concentration using Lipofectin; this method of delivery was chosen because it was more efficient than electroporation with these primary fibroblast cells, consistently giving approximately 50% transfection efficiency with an EGFP plasmid (unpublished data). At 48 h after transfection, the cells were harvested to prepare mRNA and protein samples. The 3813 cells treated with the control ASO 00–00 or buffer alone gave 31% exon 7 inclusion. After treatment with ASO 07–21 or 34–48, the apparent level of exon 7 inclusion increased to 58% and 61%, respectively (Figure 8A, left); based on the standard deviations (unpublished data; *n* = 3), this corresponds to a 1.9 ± 0.04 fold increase for ASO 07–21, and a 2.0 ± 0.04 fold increase for ASO 34–48, relative to the treatment with the control ASO 00–00. The increase in the percentage of exon 7 inclusion could potentially reflect reduced exon skipping alone, without the desirable concomitant increase in exon inclusion. To rule out this possibility, we used *GAPDH* mRNA as a loading control, and calculated the ratio of SMN full-length mRNA to *GAPDH* mRNA, normalized to that observed with the control ASO 00–00 (Figure 8A, right). The ratio increased by approximately 1.6-fold in ASO 07–21-treated cells and approximately 1.8-fold in ASO 34–48-treated cells. To detect changes at the protein level, we used α-tubulin as a loading control. The normalized ratio significantly increased by approximately 1.8-fold and approximately 2.1-fold in ASO 07–21- and ASO 34–48-treated samples, respectively, compared to ASO 00–00 (Figure 8B). Assuming a delivery efficiency of approximately 50%, the actual effects should be approximately 2-fold greater in the cells that took up the ASOs.

**Figure 8:**
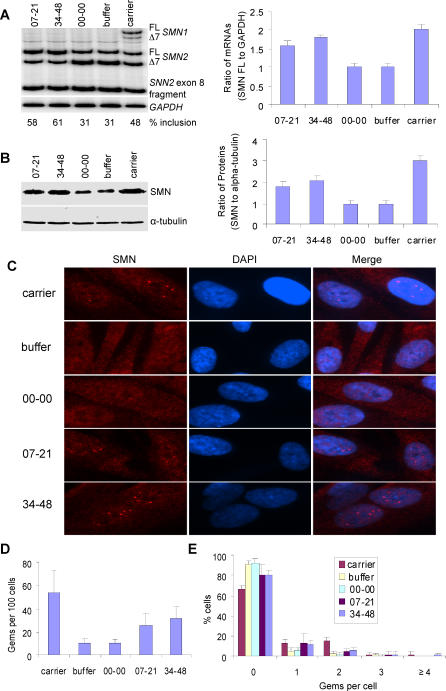
Effect of ASOs 07–21 and 34–48 in SMA Type I Patient 3813 Fibroblasts Endogenous *SMN2* mRNA, SMN protein levels, and gem number were analyzed after each ASO at a concentration of 100 nM was transfected into patient fibroblasts using Lipofectin. Two days later, RNA or protein samples were collected, or indirect immunofluorescence microscopy was carried out. Carrier 3814 fibroblasts were used as a positive control, and ASO 00–00 and buffer treatments were used as negative controls. (A) *SMN2* mRNA was analyzed by radioactive RT-PCR, using GAPDH mRNA as a loading control. The percent of *SMN2* exon 7 inclusion in each lane is shown below the autoradiogram. (B) SMN protein was detected by Western blotting, and the same blot was re-probed with antibody against α-tubulin as a protein-loading control. The histograms on the right of (A) or (B) show the corresponding quantitation from three independent experiments; the ratios are normalized to that observed with the buffer control, and error bars show the standard deviations. (C) Effect of ASOs 07–21 and 34–48 on nuclear gems. Nuclei were counterstained with DAPI. Examples of treated cells with two or more gems are shown. (D and E) Both the total number of gems per 100 cells (D) and the number of cells with multiple gems (E) significantly increased after treatment with ASOs 07–21 or 34–48.

Gems are nuclear bodies in which SMN protein accumulates [4]. A correlation between the number of gems in patient-derived fibroblasts and SMA clinical severity has been demonstrated [41]. To assess the effects of ASOs 07–21 and 34–48 on nuclear gem counts, we used indirect immunofluorescence with an anti-SMN monoclonal antibody, after transfection of 3813 cells with either ASO. The cells treated with the unrelated control oligonucleotide contained an average of 10 gems per 100 cells; however, when the cells were treated with ASO 07–21 or 34–48, the average gem number increased to 26 or 31 per 100 cells, respectively. The carrier 3814 fibroblast cells had approximately 50 gems per 100 cells (Figure 8C–8E). In addition, after treatment with ASO 07–21 or 34–48, the number of cells containing multiple gems increased, and the number of cells containing no gems decreased. These results confirm that ASOs 07–21 and 34–48 significantly promote full-length SMN protein expression.

## Discussion

Multiple strategies aimed at correcting exon 7 splicing of *SMN2,* a modifying gene for SMA, have been investigated. Mechanistic studies to understand how *SMN2* exon 7 is alternatively spliced led to the discovery of several *cis*-elements and *trans*-acting factors that can be targeted to stimulate exon 7 inclusion [42]. A number of drugs, including synthetic compounds that can modify *SMN2* splicing, have been identified using cell-based high-throughput drug screening [42]. On the basis of the current knowledge of splicing mechanisms, we and others have employed antisense-based technologies to stimulate exon 7 inclusion. In recent years, a growing number of studies have demonstrated that MOE-modified ASOs (with a phosphodiester or a phosphorothioate backbone) and antisense PNAs can be valuable tools, not only for dissecting gene function, but also for clinical applications [19]. Our previous work showed that ESSENCE compounds have the potential to treat diseases caused by exon skipping resulting from the loss of ESEs in mutant genes, including *SMN2* [34]. A critical parameter in ESSENCE effectiveness is the selection of the optimal binding site along a target exon. In principle, the best target sequences are those that comprise negative exonic splicing signals or secondary structures, such as ESSs.

In this study, we systematically analyzed *SMN2* exon 7 with a large number of MOE ASOs to identify putative ESSs. We employed a two-step ASO walk method, with an initial coarse ASO walk in 5-nt steps along the entire exon, followed by high-resolution single-nucleotide walks within the regions identified in the first step. With the first-step walk, we identified two ASO targets of potential therapeutic importance. The second-step microwalks optimized the ASOs and defined the apparent boundaries of the two ESS-containing regions. Using three independent splicing assays, we identified two potent ASOs that have significant therapeutic potential for SMA treatment. Our data also suggest that the two-step ASO walk is a powerful general method that can be used for screening inhibitory or stimulatory splicing regions present in the target exon(s) and the surrounding intron sequences of any gene.

Our data revealed an essential core sequence in the center of the 54-nt exon 7 from +22 to +33 (GAAGGAAGGTGC), which is surrounded by two separate inhibitory regions (A and B) containing negative splicing signals, with region A extending close to the upstream 3′ splice site (+4 to +21) and region B extending close to the downstream 5′ splice site (+34 to +51) (Figure 9). Blocking any part of the central core sequence promoted efficient skipping of exon 7, whereas blocking either of the two inhibitory regions promoted exon 7 inclusion. The 5′ portion of the central core sequence comprises a previously identified Tra2β1-binding motif (GAAGGA) [38]; the 3′ part (AGGTGC) may represent another *cis*-acting element that is also crucial for exon 7 recognition. A relatively long conserved sequence in the middle of exon 7, surrounded by two short inhibitory sequences, was previously identified by an iterative in vitro selection method [43]. Through an analysis of mutability, three segmental sequences with negative or positive *cis*-acting elements were inferred: the most conserved residues are located in the middle and form a conserved tract (+16 to +44), whereas the highly mutable upstream nucleotides form an inhibitory region (+3 to +15), and seven highly mutable downstream nucleotides (+45 to +51) form another negative element [43]. Our data sharpen the boundaries for these three segments, as verified by three different splicing assays and several ASOs, particularly the two most effective ASOs, 07–21 and 34–48. The target sequence of the 15-mer ASO 07–21 overlaps by six nucleotides with the previously reported conserved tract, and 11 out of 15 target nucleotides of ASO 34–48 are part of this conserved track. Nucleotides A36 and C37, which fall within the inhibitory region B, have been suggested to be part of a stimulatory motif, as the double mutation A36U/C37U abolishes exon 7 inclusion in the *SMN1* gene [43]. However, the double mutation might create a stronger inhibitory motif, or strengthen a secondary RNA structure that impairs exon 7 recognition.

**Figure 9:**
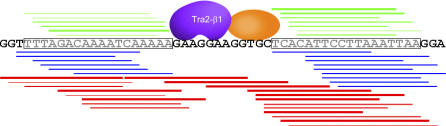
Schematic Diagram of the In Vivo Effects of All Tested ASOs on Exon 7 Inclusion An essential core sequence (in bold) surrounded by two inhibitory regions, A and B (boxed), in *SMN2* exon 7. Horizontal bars represent ASOs with stimulatory effects (green), inhibitory effects (red), or neutral effects (blue). The thicker the bars, the stronger the effects. Tra2β1 and another putative activator are shown bound to the central core region.

We searched exon 7 for putative ESSs with the Web servers PESX (http://cubweb.biology.columbia.edu/pesx) and ACESCAN2 (http://genes.mit.edu/acescan2/index.html). PESX found no ESS motifs, whereas ACESCAN2 found three putative ESS motifs: GGTTTT (+1 to +6), TTTTAG (+3 to +8) and TTCCTT (+39 to +44). The first two motifs partially overlap with the inhibitory region A, and the last motif resides within the inhibitory region B, so these motifs might contribute to the inhibitory properties of regions A and B.

We note that both optimized ASOs are 15-mers, whereas all five of the 18-mer ASOs (36–53, 35–52, 34–51, 33–50, and 32–49) displayed more or less inhibitory effects on *SMN2* exon 7 inclusion. The longer 18-mer ASOs are likely to overlap two or more binding sites, resulting in complex effects. The strong inhibitory effect of ASO 32–49 and the relatively weak inhibitory effect of ASO 33–50 might be explained by their interference with the positive regulation conferred by the essential core sequence, because the 5′-most boundary nucleotide of their target sequence is 33G and 34C, respectively. The other three ASOs, whose target sequences lie next to the exon–intron junction, might interfere with recognition of the 5′ splice site.

A small fraction of the ASOs had inconsistent effects on exon 7 splicing in the different splicing assays. One type of inconsistency occurred between the minigene splicing assays (both in vitro and in vivo) and the endogenous gene assay. The other type of inconsistency occurred between the in vitro splicing assay and the in vivo splicing assays. The first type of inconsistency may reflect the differences in the pre-mRNA substrates, although *SMN2* exon 7 was identical in all the assays. In both the in vitro splicing assay and the in vivo minigene splicing assay, a small minigene was used. Higher-order structure differences between the small minigene pre-mRNA and the much larger endogenous pre-mRNA might affect the accessibility of some ASOs to their target sequences in exon 7, resulting in splicing differences. The second type of inconsistency might reflect differences in the splicing-reaction environments between the cell-free splicing assay and the cell-based splicing assays, even though in general, the in vitro splicing reactions accurately reproduce cellular splicing events. For example, it is possible that the effects of some ASOs are more sensitive to changes in the concentration of certain splicing factors resulting from the nuclear extraction procedure. Another possibility is that the discrepancies result from kinetic differences and the coupling between transcription and mRNA processing that normally occurs in vivo, but not in standard in vitro splicing reactions [44]; moreover, transcription from different promoters—as in the minigene and the endogenous gene—can also affect use of alternative cassette exons in vivo [45].

Why ASOs 07–21 and 34–48 are more effective than the original ones, which bind to their target sequences only one or two nucleotides upstream or downstream, is currently under investigation. Because the ASOs have the same lengths and the same or similar G/C contents, annealing kinetics is probably not the cause, assuming equal accessibility to their target sites in exon 7. Two possible explanations of the effects of ASOs 07–21 and 34–48 are that they precisely block one or more splicing silencers, or efficiently disrupt an inhibitory RNA secondary structure. It has been reported that hnRNP A1 can inhibit *SMN2* and *SMN1* exon 7 inclusion [12,13]. In region A of *SMN2,* SF2/ASF presumably fails to block the cooperative propagation of hnRNP A1, due to the loss of an SF2/ASF motif caused by the C6T transition [13]. ASO 07–21 binds a 15-nt sequence that spans the mutant SF2/ASF motif; therefore, it is reasonable to assume that one of the roles played by ASO 07–21 is similar to a proposed function of SF2/ASF in *SMN1* exon 7, i.e., blocking the propagation of hnRNP A1. ASO 07–21 might also block other inhibitory motifs residing in region A, for which an extended inhibitory context has been proposed [46]. We have unexpectedly observed in microwalk A that the 12-mer ASO 04–15 inhibited exon 7 inclusion; ASO 09–20 was likewise inhibitory, at least in vivo. These results suggest that the inhibitory effect of region A is the result of interplay among multiple *trans*-acting factors. It is likely that ASOs 04–15 and 09–20 can effectively block an undefined weak stimulatory signal, but cannot efficiently block the propagation of hnRNP A1 and possibly other repressors. A terminal-stem loop has been predicted in the less-studied region B, extending to two intron 7 nucleotides [47]. Though it is possible that the inhibitory property of region B is attributable to the proposed RNA secondary structure itself, other possibilities cannot be ruled out, including the existence of one or more splicing repressors that recognize a specific motif or RNA structure. Additional experimental studies will be required to elucidate the precise splicing-stimulatory mechanism of ASO 34–48.

We cannot exclude the possibility that the stimulatory ASOs act in part by stabilizing the pre-mRNAs and/or mRNAs to which they hybridize. Because only some of the exon 7 ASOs stimulate exon 7 inclusion, such a stabilization effect would presumably involve masking of specific instability elements. However, the effect of the ASOs is primarily, if not exclusively, at the level of splicing, because an effect at the level of mRNA half-life should result in the same proportional increase in *SMN1* and *SMN2* full-length mRNAs, yet the effects on *SMN1* were much smaller, consistent with a switch in alternative splicing from the low basal level of exon 7–skipped mRNA. In addition, we did not observe any ASO-mediated stabilization of the labeled pre-mRNA in the in vitro splicing experiments.

We used three different assays to demonstrate that the ASOs that promote exon inclusion are compatible with synthesis of full-length SMN protein, indicating that they do not block mRNA export or translation. First, we showed that ASOs 07–21 and 34–48 promote efficient expression of reporter proteins corresponding to exon 7 inclusion in HEK293 cells. Second, we showed that the same ASOs promote expression of full-length SMN protein in primary fibroblasts from a type I SMA patient; though significant, this effect appears subtle because of the relatively low transfection efficiency with these cells. Finally, we showed that the same ASOs promote an increase in the number of nuclear gems detected with anti-SMN antibody in the patient fibroblasts; this single-cell immunofluorescence assay obviates the problem of transfection efficiency and provides an indirect readout for SMN protein levels [4,41].

Our initial goal in this study was to define optimal antisense targets to maximize ESSENCE effectiveness. However, our optimized antisense targets should also be useful with bifunctional ASOs [30,31]. The antisense portions of the bifunctional ASOs used in two earlier studies were not optimized. The first study used a 15-mer ASO targeting the exon 7 sequence +2 to +16 [30], i.e., the same target sequence as that of our ASO 02–16. However, we found that this ASO had a slightly negative effect on exon 7 inclusion in vivo with both the *SMN2* minigene and the endogenous *SMN2* gene, though it had a slightly stimulatory effect on splicing in vitro (Figures 3B, 5A, and 5B). ASO 02–16 was one of few ASOs that gave inconsistent effects among the different splicing assays we used. The second study used a 20-mer ASO targeting the exon 7 sequence +6 to +25 [31], which partially overlaps the central core sequence that is essential for exon 7 inclusion. On the basis of our results, we predict that more-effective bifunctional ASOs targeting exon 7 can be designed, using the ASO 07–21 or 34–48 sequences as the antisense moiety.

Although we expect that even more-potent effectors can be designed by attaching ASOs 07–21 or 34–48 to activation-domain peptides or SR protein–binding sites—as in the ESSENCE [34] and TOES [29] methods—these ASOs appear to be sufficiently effective on their own to be tested in animal models of SMA. ASOs with the same chemistry were recently demonstrated to be active modulators of splicing in mice [48]. More generally, the antisense walk strategy described here should be applicable to identifying exon-targeting ASOs that modulate alternative splicing of normal or mutant genes. At the same time, these ASOs can facilitate the identification of important *cis*-regulatory sequences that can be further investigated to elucidate splicing mechanisms, and to identify *trans*-acting factors that may be useful molecular targets for disease therapy.

## Materials and Methods

### Oligonucleotide synthesis.

Synthesis and purification of chimeric 2′-*O*-methoxyethyl–modified oligonucleotides with a phosphodiester backbone were performed using an Applied Biosystems 380B automated DNA synthesizer (Applied Biosystems, Foster City, California, United States) as described previously [49]. The oligonucleotides were dissolved in water. The sequences of all the oligonucleotides are indicated in Table I.

### Constructs.

SMN minigene constructs used for both the in vitro splicing assay and the in vivo minigene splicing assay in HEK293 cells were pCI-SMN1 and pCI-SMN2, which are derivatives of pCISMNxΔ6-WT and pCISMNxΔ6-C6T, respectively [11,50]. The *SMN* minigenes comprise the 111-nt–long exon 6, a 200-nt shortened intron 6, the 54-nt exon 7, the 444-nt intron 7, the first 75 nt of exon 8, and a consensus 5′ splice site (CAGGTAAGTACTT) to promote exon definition in vitro. We amplified the minigenes with primer set T7-F2 (5′-TAC TTA ATA CGA CTC ACT ATA GGC TAG CCT CG-3′) and SMN8-75-5′R-SalI-NotI (5′-GAA GCG GCC GCG TCG ACA AGT ACT TAC CTG TAA CGC TTC-3′), and inserted them into vector pCI-neo (Promega, Madison, Wisconsin, United States) using XhoI and NotI. Two restriction sites, SalI and NotI, were included in the reverse primer. Four minigene constructs were used for protein expression: constructs pEGFP-SMN2 and pEGFP-SMN1 have the same minigenes as in pCI-SMN2 and pCI-SMN1, but without the consensus 5′ splice site. Minigene inserts were amplified with primer set Xho-HA-E6-F (5′-GAT CTC GAG AGT ACC CAT ACG ACG TAC CAG ATT ACG CTA TAA TTC CCC CAC CAC CTC CC-3′) and Pr-E8–75-BamH-R (5′-CGG GAT CCT AAC GCT TCA CAT TCC AGA TC-3′), digested with XhoI and BamHI and subcloned into the XhoI and BamHI sites in pEGFP-C1 (Clontech, Mountain View, California, United States). Constructs pEGFP-SMN2Δ6 and pEGFP-SMN1Δ6 are similar to pEGFP-SMN2 and pEGFP-SMN1, but most of exon 6 was deleted, with only the last nine nucleotides retained to keep the natural context of the 5′ splice site of exon 6. Minigene inserts were amplified with Pr-HA-I6-Xho-F (5′-ATC TCG AGA GTA CCC ATA CGA CGT ACC AGA TTA CGC TTA TTA TAT GGT AAG TAA TCA CTC-3′) and Pr-E8–75-BamH-R, and were subcloned into XhoI and BamHI double-digested pEGFP-C1. One copy of the HA epitope tag sequence was included in primers Xho-HA-E6-F and Pr-HA-I6-Xho-F, so that all protein products expressed from the four EGFP-fused minigene constructs have an N-terminal HA epitope tag and can be detected with anti-HA antibody.

### In vitro splicing.

To generate in vitro splicing substrates, we linearized plasmids pCI-SMN1 and pCI-SMN2 with SalI, and transcribed them with T7 RNA polymerase (Promega) in the presence of α-^32^P-UTP and ^7Me^GpppG cap analog [11,51]. Substrates were purified by denaturing polyacrylamide gels and spliced in HeLa cell nuclear extract, as described [52]. We incubated 8 fmol of transcript in 10-μl splicing reactions containing 3 μl of nuclear extract and 1.6 mM MgCl_2_. After incubation at 30 °C for 4 h, we extracted the RNA and analyzed it on 8% denaturing polyacrylamide gels, followed by autoradiography and phosphorimage analysis with an Image Reader FLA-5100 (FujiFilm Medical Systems, Stamford, Connecticut, United States). We calculated exon 7 inclusion as a percentage of the total amount of spliced mRNA, i.e., included mRNA × 100/(included mRNA + skipped mRNA). The signal intensity of each mRNA isoform band was normalized according to its U content.

### Cell culture and transfection.

HEK293 cells, SMA type I homozygous and carrier fibroblasts (3813 and 3814; Coriell Cell Repositories, Camden, New Jersey, United States) were cultured in Dulbecco's modified Eagle's medium (DMEM; Invitrogen, Carlsbad, California, United States) containing 10% (v/v) fetal bovine serum and antibiotics (100-U/ml penicillin G and 100-μg/ml streptomycin). For transfection of MOE oligonucleotides and plasmids into HEK293 cells, electroporation was used, followed by puromycin selection (Figures 2 and 5–7). Briefly, 90 μl of freshly-prepared cells (1 × 10^7^/ml) suspended in Opti-MEM medium was mixed with 10 μl of ASO/DNA mixture in a 1-mm cuvette. A total of 2.5 μg of the plasmid pBabe Puro [53] was included in the ASO/DNA mixture for selection. For the in vivo minigene splicing assay and the minigene protein assays, 5 μg of each minigene plasmid was used. For electroporation, 80 volts and 500 μF were applied with a Gene Pulser II apparatus (Bio-Rad, Hercules, California, United States). Shocked cells were then cultured in 60-mm dishes in normal medium. Untransfected cells were killed by treatment with 2-μg/ml puromycin for 20 h. For transfection of MOE oligonucleotides into 3813 cells, Lipofectin (Invitrogen) was used according to the manufacturer's instructions.

### RT-PCR.

Total RNA was isolated with Trizol reagent (Invitrogen), and 2 μg of each RNA sample was used per 20-μl reaction for first-strand cDNA synthesis with Oligo-dT and Super Script II reverse transcriptase (Invitrogen). Splicing products were amplified semi-quantitatively using 22 PCR cycles (94 °C for 30 s, 55 °C for 30 s, and 72 °C for 36 s). Primer set T7-F2 (see above) and E8–75+5′R (5′-AAG TAC TTA CCT GTA ACG CTT CAC ATT CCA GAT CTG TC-3′) was used to amplify minigene pCI-SMN2 transcripts. Primer set EGFP-C (5′-CAT GGT CCT GCT GGA GTT CGT G-3′) and Pr-E8–75-BamH-R was used to amplify transcripts from plasmids pEGFP-SMN1, pEGFP-SMN2, pEGFP-SMN1Δ6, and pEGFP-SMN2Δ6. Primer set E3-F (5′-ACT TTC CCC AAT CTG TGA AGT A-3′) and E8-R (5′-CAT TTA GTG CTG CTC TAT GCC AGC-3) was used to amplify endogenous *SMN2* transcripts in 3813 cells; the GAPDH mRNA in 3813 cells was used as an internal control, and was amplified with primers GAPDH-F (5′-AAG GTG AAG GTC GGA GTC AAC GG-3′) and GAPDH-R (5′-CCA CTT GAT TTT GGA GGG ATC TC-3′). Primer set E6-F (5′-ATA ATT CCC CCA CCA CCT CCC-3′) and E8–467 (5′-TTG CCA CAT ACG CCT CAC ATA C-3′) was used to amplify endogenous *SMN1/2* transcripts in HEK293 cells, and the PCR products were then digested with DdeI to distinguish their origin *(SMN1* or *SMN2).* All PCR products were labeled with α-^32^P-dCTP and separated on 6% or 8% native polyacrylamide gels. The extent of exon 7 inclusion was calculated as above, and the signal intensity of each cDNA band was normalized according to its G+C content.

### Plotting dose-response curves and kinetic curves.

All curves were plotted with Prism software (Graphpad Software, http://www.graphpad.com). To compare the effects of ASOs 07–21, 34–48, 06–20, and 36–50 on exon 7 inclusion in vitro, sigmoidal curve-fitting was used, with X values plotted on a logarithmic scale. The EC50 value of each ASO was calculated by the software. Sigmoidal curves were also plotted for the effects of ASOs 07–21 and 34–48, respectively, on endogenous *SMN* genes in HEK293 cells. The time-course analysis of ASOs 07–21 and 34–48 in HEK293 cells was plotted using exponential-decay curve-fitting.

### Western blotting.

Two or 3 d after transfection, HEK293, 3813, or 3814 cells were harvested and lysed with lysis buffer (50 mM Tris, [pH 7.6], 150 mM NaCl, 2 mM EDTA, 1.0% Nonidet P-40, 1 mM PMSF), supplemented with protease inhibitor cocktail (Roche Applied Science, Indianapolis, Indiana, United States). Protein samples were separated by SDS-PAGE (sodium dodecyl sulfate -polyacrylamide gel electrophoresis) and transferred to nylon membranes. The blots were probed with monoclonal mouse anti-SMN (BD Transduction Laboratories; BD Biosciences, San Jose, California, United States), monoclonal mouse anti-HA (HA.11; BAbCO, Richmond, California, United States), or monoclonal mouse anti–α-tubulin (Sigma, St. Louis, Missouri, United States) antibody, followed by secondary goat anti-mouse antibody labeled with yellow-fluorescent Alexa Fluor 532 dye (Molecular Probes/Invitrogen). The signal was detected with an Image Reader FLA-5100 (FujiFilm Medical Systems).

### Immunofluorescence and gem counting.

The 3813 and 3814 fibroblasts were plated on coverslips and grown in DMEM. After overnight growth, the cells were transfected with 100 nM oligonucleotides. At 48 h post-transfection, the cells were washed with phosphate-buffered saline (PBS) and fixed with 3.7% paraformaldehyde in PBS for 20 min. After washing with PBS, the cells were permeabilized in 0.1% Triton X-100 in PBS for 3 min, blocked for 30 min in blocking buffer (5% goat serum, 3% IgG-free bovine serum albumin [BSA]), and incubated for 3 h in blocking buffer containing mouse monoclonal anti-SMN antibody. The cells were then washed three times with PBS and incubated for 2 h in blocking buffer containing goat anti-mouse secondary antibody Alexa Fluor 594 (Molecular Probes/Invitrogen). The cells were again washed with PBS three more times, and coverslips were mounted with Prolong Gold mounting solution containing DAPI (Molecular Probes/Invitrogen) for nuclear staining. Cells were analyzed using an Axioplan 2i fluorescence microscope (Carl Zeiss, Thornwood, New York, United States) equipped with Chroma filters (Chroma Technology, Rockingham, Vermont, United States). OpenLab software (Improvision, http://www.improvision.com) was used to collect digital images from a CCD camera (Hamamatsu, Bridgewater, New Jersey, United States). To count gems, first a field was randomly selected using the DAPI channel, the cell number was determined based on the DAPI counterstaining, and then the gems were counted in the red channel. At least three different fields were counted, and each field contained more than 40 cells.

## Supporting Information

### Accession Numbers

The UniProt (http://www.pir.uniprot.org) accession number for SMN is Q16637–1. The Entrez Gene (http://www.ncbi.nlm.nih.gov/entrez/query.fcgi?db=gene) GeneID numbers for the genes discussed in the paper are *SMN1* (6606) and *SMN2* (6607).

## Abbreviations

ASO: antisense oligonucleotide
EGFP: enhanced green fluorescent protein
ESE: exonic splicing enhancer
ESS: exonic splicing silencer
ESSENCE: exon-specific splicing enhancement by small chimeric effectors
HA tag: hemagglutinin epitope tag
hnRNP: heterogeneous nuclear ribonucleoprotein
nt: nucleotide
PBS: phosphate-buffered saline
RNP: ribonucleoprotein particle
SF2/ASF: splicing factor 2/alternative splicing factor
SMA: spinal muscular atrophy
SMN: survival of motor neuron
SR: serine/arginine-rich protein
Tra2β1: transformer 2 beta 1 protein
